# Undetectable thyroglobulin makes ^123^I whole-body scan and stimulated thyroglobulin obsolete in follow-up care of differentiated thyroid cancer: a retrospective study

**DOI:** 10.1186/s13044-021-00114-0

**Published:** 2021-10-19

**Authors:** Bastiaan Sol, Bert Bravenboer, Brigitte Velkeniers, Steven Raeymaeckers, Marleen Keyaerts, Corina Emilia Andreescu

**Affiliations:** 1 Department of Endocrinology, UZ Brussel, Laarbeeklaan 101, 1090 Brussels, Belgium; 2 Department of Radiology, UZ Brussel, Laarbeeklaan 101, 1090 Brussels, Belgium; 3 Department of Nuclear Medicine, UZ Brussel, Laarbeeklaan 101, 1090 Brussels, Belgium

**Keywords:** Thyroglobulin, Differentiated thyroid Cancer, Ultrasound of the neck, ^123^I whole-body scan

## Abstract

**Background:**

Differentiated thyroid cancer (DTC) is a common malignancy with increasing incidence. Follow-up care for DTC includes thyroglobulin (Tg) measurement and ultrasound (US) of the neck, combined with ^131^I remnant ablation when indicated. Diagnostic precision has evolved with the introduction of the new high-sensitive Tg-assays (sensitivity ≤0.1 ng/mL). The aim of the study was to determine the prognostic utility of high-sensitive Tg and the need for other diagnostic tests in DTC.

**Methods:**

This was a retrospective, observational study. Patients with pathologically confirmed DTC, treated with total thyroidectomy and ^131^I remnant ablation, who had their complete follow-up care in our institution were selected (October 2013–December 2018). Subjects with possible thyroglobulin autoantibody interference were excluded. Statistical analysis was performed using the IBM SPSS® Statistics 24 software package.

**Results:**

Forty patients were eligible for analysis. A total of 24 out of the 40 patients (60%) had an undetectable high-sensitive Tg 6 months after total thyroidectomy. None of these patients had a stimulated Tg above 1 ng/mL, or remnant on the ^123^I Whole-Body Scan (WBS) after 1 year of follow-up. Ultrasound of the neck, performed between 6 and 12 months postoperative, was negative in 21 out of the 24 patients.

**Conclusions:**

This study shows that an undetectable high-sensitive Tg can change the management of patients with DTC and decrease the use and need of stimulated Tg and ^123^I WBS.

## Background

Differentiated thyroid cancer (DTC) is a common malignancy, with increasing incidence every year. It was the fifth most common cancer in women in the USA in 2015, with 62.000 new cases reported [1]. The increased incidence is mostly attributed to the improved use of diagnostic imaging. Mortality rates have dropped or remained low [2]. As a result, the challenge is to avoid overtreatment, but recognize high-risk patients and possible recurrence early.

In 2015, the American Thyroid Association (ATA) published the latest guidelines on management of DTC [3]. Total thyroidectomy, with adjunctive ^131^I remnant ablation when necessary, is still considered standard of care. Thyroglobulin (Tg) measurement, in the absence of interfering Tg autoantibodies (TgAbs), together with ultrasound (US) of the neck, should be used to detect recurrent or persistent disease after treatment [3, 4]. Subsequent follow-up can include a ^123^I whole-body scan (WBS) when considered necessary [3, 5]. The advantage of a ^123^I WBS over a diagnostic ^131^I WBS is that ^123^I has no β emission, and a shorter half-life, while the diagnostic performance remains equal [6].

Serum Tg is obtained after thyroid stimulating hormone (TSH) stimulation to improve diagnostic accuracy, either following thyroid hormone withdrawal, or after injection of recombinant human TSH (rhTSH) [7]. However, thyroid hormone withdrawal may have a significant negative impact on quality of life with dangerous adverse events, especially in at-risk patients, while administration of rhTSH causes a significant financial burden for society and the patient [8]. New Tg-assays with a functional sensitivity lower than or equal to 0.1 ng/mL, the so-called high-sensitive Tg, are now being utilized in the follow-up of low -or intermediate risk patients [9, 10]. A meta-analysis of the diagnostic performance of high-sensitive Tg demonstrated a very high negative predictive value (NPV) [11].

However, there is still debate regarding the validity and utility of basal Tg (bTg) during follow-up, and many centres still consider stimulated Tg (sTg) inevitable during follow-up care of DTC [12]. The aim of this study was to review and determine the prognostic utility of bTg with the new high-sensitive Tg-assays.

## Methods

### Study group

The study was approved by the institutional review board and Medical Ethics Committee UZ Brussel. Need for written informed consent was waived. A retrospective analysis was conducted for all patients over the age of 18 who:

- had undergone a total thyroidectomy, with diagnosis of DTC on pathological examination.

- received subsequent ^131^I remnant ablation.

- had their complete follow-up care (blood analyses of bTg and sTg, US of the neck, ^131^I remnant ablation and ^123^I WBS) in our institution from October 2013 onwards, after which a more sensitive Tg assay with a functional sensitivity of 0.1 ng/ml was introduced.

Subjects without ^131^I remnant ablation, data of histopathological diagnosis, bTg and sTg measurement, or ^123^I WBS results were excluded. Subjects with possible TgAbs interference were excluded as well.

### Data

Data were retrieved from the medical records and included histopathological results, blood analyses (bTg, sTg, TSH, free Thyroxine (fT4), TgAbs), US of the neck, ^131^I and ^123^I WBS results over a period of 1 year after thyroidectomy.

bTg was measured at a 3 to 6 month interval using the Elecsys Tg II immunoassay with functional sensitivity up to 0.1 ng/mL. A value below 0.1 ng/mL was reported as undetectable. sTg is defined as the thyroglobulin concentration after stimulation. It was measured 5 days after 2 rhTSH (thyrotropin alfa, Sanofi Genzyme) injections, or 3 weeks after hormone withdrawal. A value below 1 ng/mL was reported as a low sTg. TgAbs were measured at a 3 to 6 months interval using the Elecsys Anti-Tg Assay. The lower detection limit was 10.0 mU/L. The threshold value for positivity was the proposed manufacturer’s cut-off (115 mU/L).

TSH and fT4 were measured at a 3 to 6 months interval. In accordance with the ATA guidelines, levothyroxine dosage after surgery was modified to maintain a suppressive low TSH. The specific target serum TSH was risk-dependent.

A post-operative US of the neck was performed at a 6 to 12 months interval by a certified radiologist. The ultrasound was performed on a Canon Aplio i800 system, using a dedicated thyroid program on a multi-frequency ultra-wideband linear 18 Mhz probe. The neck was scanned in the axial and sagittal plane for remnant thyroid tissue. Different colour Doppler modes were used to assess the vascularity of a suspected remnant. The different anatomical regions of the neck were scanned for atypical or enlarged lymphnodes.

Indeterminate or inconclusive results were discussed interdisciplinary and, when necessary, followed by fine needle aspiration (FNA), Computed Tomography (CT) or Magnetic Resonance imaging (MRI) of the neck.

Conventional planar WBS (^131^I and ^123^I) was combined with Single Photon Emission Computed Tomography (SPECT) to enable precise localization and characterization of abnormal foci of Radioactive iodine (RAI) accumulation.

Remnant ablation with ^131^I was performed after rhTSH stimulation in low to intermediate risk patients within 2 months after surgery. Hormone withdrawal was preferred in high risk patients. Dosage was decided multidisciplinary and ranged from 30 mCi to 150 mCi.

A diagnostic ^123^I WBS was performed 1 year after the total thyroidectomy: rhTSH (thyrotropin alfa, Sanofi Genzyme) was injected (intramuscular) on day one and two. On day five, 2 h after injection of ^123^I, planar scintigraphy was performed. Optional SPECT/CT was performed on day four.

### Analysis

The study population was first assessed for detectable vs. undetectable bTg, 6 months after total thyroidectomy. The 2 groups were correlated to the sTg at 12 months, the US of the neck after 6 to 12 months and the diagnostic ^123^I WBS after 1 year. Both groups were subsequently classified into 3 groups: a low-, intermediate-, or high-risk group depending on the tumour histology, locoregional or distant metastases, lymphovascular invasion and avidity on post therapy scan as proposed by the ATA 2015 guidelines.

### Statistical analysis

Descriptive statistics were applied to all collected variables expressed as frequencies for categorical data or mean values ± standard deviations for continuous data. Group comparisons were carried out using the chi-square test. A *p* value less than 0.05 was considered significant. Statistical analysis was performed using the IBM SPSS® Statistics 24 software package.

## Results

**Fig. 1:**
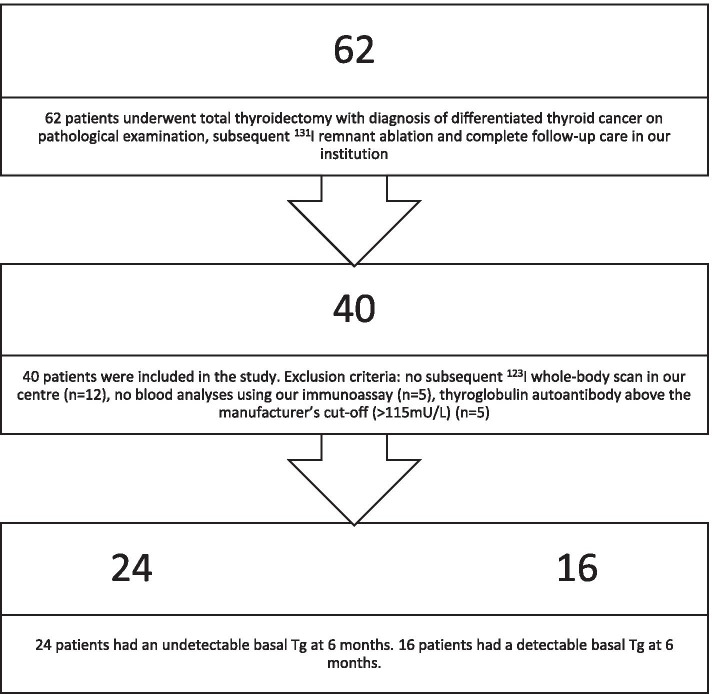
Distribution of study population

Sixty-two patients over the age of 18 had undergone a total thyroidectomy, with diagnosis of DTC on pathological examination, and received subsequent ^131^I remnant ablation, with complete follow-up care in our institution between October 2013 and December 2018. Seventeen patients were excluded from the analysis: no subsequent ^123^I WBS was performed (*n* = 12), or the blood analyses were not performed using our immunoassay (*n* = 5). An additional 5 patients were excluded because TgAbs were positive at 6 months after total thyroidectomy, with possible interference of the Tg results. In the end, 40 subjects were eligible for analysis (Fig. 1). The average age of the patients was 45 years, with a range from 25 to 70 years old. A predominant female population (75%) was observed. Average tumour size was 2.4 cm, with mainly papillary type histology (90%). Central lymph node dissection was performed in 10 patients, and lymph node invasion was confirmed in 5 patients after pathological examination. Seven tumours were larger than 4 cm. Vascular invasion was observed in 14 out of the 40 tumours. Approximately one third (32.5%) of the tumours were multifocal on pathological examination. More than one third (35%) of the surgical specimens had microscopically positive margins, and in 6 specimens extrathyroidal extension was observed. More than half (52.5%) of the patients had low risk of cancer recurrence after initial treatment. Seven patients had RAI-avid metastatic foci in the neck on the post-ablation WBS. None of the patients showed distant metastases. A total of 4 patients were classified as high risk. The reason for high risk classification was the presence of a pathological lymph node larger than 3 cm (*n* = 2), or extensive vascular invasion of a follicular thyroid cancer (*n* = 2). High risk characteristics were more present in older, male patients. Tumour size and microscopic positive margins were not associated with higher risk stratification (*p* > 0.05). A summary of the study population can be found in Table 1.

**Table 1:** Patient characteristics and histology of tumour samples

**Gender**	
- Male (n/total)	10/40 (25%)
- Female (n/total)	30/40 (75%)
**Mean age (years)**	45 ± 15
**ATA 2015 risk stratification**	
- Low (n/total)	21/40 (52.5%)
- Intermediate (n/total)	15/40 (37.5%)
- High (n/total)	4/40 (10%)
**Mean tumour size (cm ± SD)**	2.4 ± 1.3
**Lymph node dissection (n/total)**	10/40 (25%)
**T stage**	
- 1 (n/total)	15/40 (37.5%)
- 2 (n/total)	13/40 (32.5%)
- 3 (n/total)	12/40 (30%)
**N stage**	
- 0 (n/total)	35/40 (87.5%)
- 1 (n/total)	5/40 (12.5%)
**M stage**	
- 0 (n/total)	40/40 (100%)
- 1 (n/total)	0/40 (0%)
**AJCC stage**^**a**^ **(n/total)**	
- Stage 1 (n/total)	28/40 (70%)
- Stage 2 (n/total)	9/40 (22.5%)
- Stage 3 (n/total)	3/40 (7.5%)
- Stage 4 (n/total)	0/40 (0%)
**Histology**	
- Papillary (n/total)	36/40 (90%)
- Follicular (n/total)	4/40 (10%)
**Vascular invasion**	
- Yes (n/total)	14/40 (35%)
- No (n/total)	26/40 (65%)
**Multifocal tumour**	
- Yes (n/total)	13/40 (32.5%)
- No (n/total)	27/40 (77.5%)
**Microscopic positive margins**	
- Yes (n/total)	14/40 (35%)
- No (n/total)	26/40 (75%)
**Post-ablation WBS**	
- Remnant (n/total)	33/40 (82.5%)
- Metastatic foci in the neck (n/total)	7/40 (17.5%)
- Distant foci (n/total)	0/40 (0%)

^a^AJCC, American Joint Committee on Cancer

A total of 24 out of the 40 patients (60%) had an undetectable bTg 6 months after the total thyroidectomy (Fig. 1). None of these patients had a sTg above 1 ng/mL at 1 year of follow-up. The NPV of bTg for sTg, calculated by dividing the number of patients with both undetectable bTg and low sTg (< 1 ng/mL) by the total number of patients with undetectable bTg, indicated a value of 100% (*p* < 0.05). bTg remained undetectable at 12 months in all patients. Moreover, the ^123^I WBS was negative (no remnant or metastatic foci), indicating a NPV of 100% (*p* < 0.05) for the ^123^I WBS as well. US of the neck, performed at 6 months, was negative in 21 out of the 24 patients, resulting in a NPV of 87.5% (p < 0.05). In the remaining 3 patients, a possible remnant and/or adenopathy was described by the radiologist. However, in all 3 patients the US of the neck was deemed false positive as the abnormal findings could not be confirmed. However, FNA for cytological evaluation was not performed. One of the possible remnants was no longer visible at 12 months, resulting in a NPV of 91.6% for the US of the neck at 12 months.

Mean TSH after 6 months was higher than expected due to non-compliant patients with high TSH values. The median TSH was 0.87 mU/L. Table 2 summarizes follow-up results of patients with undetectable bTg at 6 months.

**Table 2:** Follow-up of patients with an undetectable bTg

	Undetectable bTg at 6 months
**Total**	24/24 (100%)
**Post-operative risk stratification**	
- Low risk (n)	15/24 (62.5%)
- Intermediate risk (n)	7/24 (29.2%)
- High risk (n)	2/24 (8.3%)
**sTg at 12 months**	
< 1 ng/mL (n)	24/24 (100%)
≥1 ng/mL (n)	0/24 (0%)
**Ultrasound of the neck at 6 months**	
No remnant (n)	21/24 (87.5%)
Indeterminate results (n)^a^	3/24 (12.5%)
Remnant (n)	0/24 (0%)
**Ultrasound of the neck at 12 months**	
No remnant (n)	22/24 (91.6%)
Indeterminate results (n)^a^	2/24 (8.4%)
Remnant (n)	0/24 (0%)
^**123**^**I WBS at 12 months**	
No remnant (n)	24 (100%)
Remnant (n)	0 (0%)
**TSH (mIU/L) at 6 months**	
- Mean ± SD	7.07 ± 31.8
- Median	0.87

^a^Possible remnant described by radiologist without confirmation

bTg was detectable in 16 patients (40%) at 6 months after total thyroidectomy. Six out of these 16 patients had low sTg levels (< 1 ng/mL) 1 year after thyroidectomy. Of these last patients, 2 (33.3%) were low risk, 3 (50%) intermediate risk, and 1 (16.7%) high risk. Negative imaging was found in 4 of these patients. In the 2 remaining patients, US of the neck or ^123^I WBS showed possible remnant.

## Discussion

Serum Tg and US of the neck are the recommended methods for follow-up of patients with DTC. Our study confirms how modern Tg assays are sensitive enough to overcome the need to stimulate Tg production in those patients who have undetectable bTg at 6 months, in the absence of TgAbs. Furthermore, our study demonstrates a high NPV for other diagnostic tests as well during the follow-up care of DTC.

False-low Tg results could result in undertreatment of patients with recurrent or residual cancer [13]. Assay interference with TgAbs can lead to false low Tg results [14]. Good clinical practice in the follow-up of DTC must therefore include a systematic evaluation of Tg in concurrence with the TgAbs. However, clear consensus on cut-off values for TgAb positivity and interference is lacking [15]. We therefore excluded patients in our retrospective study with significant TgAb positivity. The cut-off for interference was the manufacturer’s cut-off, as proposed by Dekker et al. [15].

Our study is in line with others, and confirms how undetectable serum bTg is highly predictive of absent residual disease in patients who have had total thyroidectomy with ^131^I ablation, and that these patients are at low risk for recurrence [16, 17]. Sunny et al. claimed that testing of Tg without stimulation could underestimate tumour burden [12]. However, the cut-off values for Tg were different in their study (10 ng/ml vs 0.1 ng/ml). The retrospective studies by Richard Kloos and by Cherk et al. reported recurrence of thyroid cancer in patients with virtual undetectable Tg values, but used cut-off levels up to 0.5 ng/mL and 1 ng/mL respectively [18, 19].

A high NPV (up to 98.6%) of undetectable bTg was shown by Castagna et al. Only 3 of their patients with undetectable bTg had positive US of the neck. However, to our knowledge, a false positive US of the neck was not excluded [16].

An excellent response and disease-free status is defined by the ATA guidelines of 2015 with negative imaging using ultrasound of the neck or ^123^I WBS, together with low -or undetectable Tg (< 0.2 ng/mL) or low sTg (< 1 ng/mL) during follow-up. Imaging is required to demonstrate an excellent response. A ^123^I WBS is still considered in the guidelines to detect RAI-avid metastatic disease in intermediate -and high risk patients, independent of bTg level [3, 5].

Our study found a NPV of 100% for the ^123^I WBS 1 year after the total thyroidectomy and ^131^I remnant ablation when bTg was undetectable at 6 months. This was independent of histological tumour type or initial ATA risk classification. However, the limited amount of intermediate  and high risk patients included could be a confounding factor.

A NPV of 87.5% for US of the neck was found when bTg was undetectable at 6 months. The remaining 22.5% were deemed false positive results. A high false-positive rate for US of the neck is in consistence with the retrospective study of Verburg et al., who showed that low or undetectable bTg could obviate the use of US of the neck in follow-up of DTC after total thyroidectomy and ^131^I ablation, since a bTg level lower than 1 µg/L was associated with considerable false-positive findings with extremely low rates of true-positive results [20].

Whenever bTg is detectable at 6 months, but sTg is < 1 ng/mL after 1 year, imaging may be obsolete as well. Following initial successful treatment, serum Tg may continue to be detectable for well over a year, and this probably relates to the biological response of DTC cells to the treatment [21].

Trimboli et al. concluded that low -or intermediate-risk patients could be considered free of disease, without further imaging, when bTg is below 0.28 ng/mL at 29 months of follow up [22]. Our study indicates that patients with an undetectable bTg at 6 months can be considered free of disease at 1 year. When bTg is detectable, a sTg can be analysed 6 months later since negative results (sTg < 1 ng/mL) may obviate further imaging as well.

Limits to our study include a retrospective analysis, the small study population, and the limited follow-up of 1 year. Many patients were excluded since they had their thyroidectomy in our centre, but their follow-up care elsewhere. Further research with larger cohorts is necessary to provide further evidence.

## Conclusions

This retrospective study further indicates the high applicability and prognostic value of high-sensitive bTg after total thyroidectomy and ^131^I remnant ablation in the absence of TgAbs. Undetectable bTg 6 months after total thyroidectomy and ^131^I remnant ablation has a NPV of 100% for the ^123^I WBS and sTg after 1 year.

The results of the present study indicate how excellent response might be identified without further imaging or invasive testing. Research with larger cohorts is necessary to provide further evidence of our hypothesis.

## Data Availability

The datasets used and/or analysed during the current study are available from the corresponding author on reasonable request.

## Abbreviations

DTC: Differentiated thyroid cancer
ATA: American Thyroid Association
Tg: Thyroglobulin
US: Ultrasound
WBS: Whole-body scan
RAI: Radioactive iodine
TSH: Thyroid stimulating hormone
rhTSH: recombinant human TSH
NPV: Negative predictive value
BTg: Basal thyroglobulin
sTg: Stimulated thyroglobulin
Tgabs: Thyroglobulin antibodies
FNA: Fine needle aspiration
CT: Computed tomography
MRI: Magnetic resonance imaging
SPECT: Single photon emission computed tomography
fT4: free Thyroxine
